# Practical Medicine

**Published:** 1880-06

**Authors:** 


*Collaborators:*

DR. L. W. CASE, DR. R. TILLEY.

### PRACTICAL MEDICINE.

DEFORMITIES OF THE VULVA PRODUCED BY DEFLORATION, MASTURBATION, SAPHISM AND PROSTITUTION.—Dr. Martineau. (*Le Praticien* and *Gazette Obstét.*, March 20, 1880, p. 87.)

Dr. Martineau has recently given a series of lectures at the Lourcine hospital, on this subject, too little known by physicians, and generally excluded from didactic works; we extract a few passages.

The external genital organs of women, as the result of their functions, of vicious habits, or of pathological lesions, undergo modifications in their aspect, and suffer deformities, which put the practitioner in the way of determining the lesions which affect them, and are to him the source of numerous indications. These deformities may be seen in young girls, in married women, as well as in prostitutes addicted to the most abject practices.

Before entering upon a description of vulvar deformities, Dr. Martineau describes the anatomy and normal aspect of the genital parts in the little girl, the girl at puberty, the married woman, etc. In the little girl the vulva is vertical, and its opening looks directly forward. It is partly open at the upper portion, and separation of the labia majora allows a view of the clitoris, which appears prominent, and the orifice of the meatus urinarius; it is closed, on the contrary, at its lower portion. An opposite condition is seen in girls at puberty; a disposition still more appreciable in women after the first sexual relations. The vulva is directed obliquely from above downwards, and from before backwards. The separation of the labia majora is slight above, and finally disappears completely, so that the clitoris and meatus are totally concealed ; on the contrary, the vulva is partly open at its inferior portion. The amount of hair seems to be in proportion to the complete development of the internal genital organs. The hairs are sometimes rare and disseminated in women in whom the uterus has not acquired its normal development, and who are in consequence sterile. (The importance given to this sign by the Jewish law to determine the nubile age of women is well known. According to the precepts of the Talmud, the woman shall have at least two hairs upon the vulva to be reputed nubile.)

Deformities of the vulva are the result of *violent defloration* or of divers vicious habits : *masturbation* and *saphism*, or of *prostitution*.

The deformities seen after violent defloration are all the more characteristic in that they are produced in younger subjects. They are principally seen in girls from 6 to 15 years of age. According to Tardieu and Toulmonche, fifty-nine times the deformities existed in girls under 11 years ; thirty-two times from 11 to 15 ; four times from 15 to 20 ; once in a maiden of 41 years. At Lourcine I find every day that these deformities may be produced at any age, and that they are as characteristic in adult women as in little girls. Sometimes they are the result of a disproportion of the genital organs, which renders the intromission of the male member difficult and impossible. Sometimes they result from imperforate vagina, from the presence of vertical bridles, from a vicious disposition of the pelvis or inferior limbs. Finally, I might mention the height of the perineum, by virtue of which, in certain women, the fourchette seems to be carried toward the pubis.

These deformities consist, in the girl, in a premature development of the sexual parts, a precocious nubility, and especially the formation, at the expense of the vulvar canal, of a sort of infundibulum, more or less wide and deep, capable of receiving the extremity of the penis, and very analogous to that which has been indicated at the anus as characteristic of pederasty. When this infundibulum exists, the physician, in practicing the vaginal touch, has the sensation of a veritable sexual canal, preceding the true vaginal canal. The hymen is generally ruptured, but at times, however, it is found at the bottom of the infundibulum, still preserving its semilunar form, and slightly lacerated on one of its borders. Such are the typical characters of deformity of the external genital organs produced by incessant and *sometimes unfruitful* efforts of the defloration.

Deformities produced by the three other causes are met with in two-thirds of the women who frequent the Lourcine. The vulvar deformities due to masturbation are not the appanage of women of gallantry; they are encountered in all classes of society. This practice also gives rise to various troubles in the organism, bearing upon the nervous system and upon nutrition. It plays, moreover, in the pathogeny of uterine affections, a rôle which should not be disregarded; to it a good many cases of constitutional metritis owe their existence. Moreover, masturbation retards their termination, and is a frequent cause of relapses and recurrences. Masturbation, in women, is the ordinary result of a slight friction of the clitoris with the finger, with the penis, or with the tongue. In the latter case it has received a particular name—saphism or tribadism. Sometimes, also, it is produced by the rubbing together of the thighs, strongly pressed one upon the other.

The deformations are sustained by the clitoris and the labia minora. They consist especially in modifications of structure resulting from the habitual congestion of which these organs become the seat. On the clitoris they affect the sheath and glans clitoris; the clitoris takes on an exaggerated development; its length, an average of (1.1 inch) 3 centimeters, may become doubled. Parent-Duchatel, in his work on "Prostitutes in Paris," mentions three prostitutes in whom the clitoris had a remarkable development; in one of them it had a length of about (3 inches) 8 centimeters, and in volume it equaled the index finger. At the same time, the glans clitoris is swollen, red, violaceous; it extends beyond the sheath—sometimes is entirely procident. The sheath is loose, long, wrinkled, forming a series of folds; its anterior extremity is detached from the glans clitoris, and leaves it uncovered.

The labia minora are elongated, more voluminous, lax, pendant, triangular; their rose color has disappeared, and they have become brown, grey, or slate-color; at the same time there is seen upon their internal face small yellow prominent points; the sphincter of the bladder may be dilated to such a degree as to produce incontinence of the urine, and I am inclined to believe that a good many cases of incontinence of the urine in girls, and even in women, have for their cause masturbation.

When masturbation is produced by friction of the thighs against each other when crossed, certain peculiarities are observed; the sheath is relatively but little developed, compared with the volume which the glans attains; on the contrary, the latter is hard, voluminous, prominent, and has a club-shaped extremity. The labia minora are also less developed than in other cases.

It would be interesting to know if the third manner of masturbation—saphism or tribadism—produced upon the vulva as characteristic deformations as the preceding. This unnatural practice is nowadays one of the most frequent in all classes of society, as well among married women as in prostitutes.

Saphism is very much in honor among the patients who frequent the Lourcine hospital, but it is difficult to determine exactly its characters, for it is always combined with handling. In order to make this study complete and satisfactory, it would be necessary to observe women united in some numbers, such as those who live in Oriental harems; it is indeed known that saphism is very frequent among these women. However it may be, in women I have examined, who have admitted this practice, it appeared to me that the border of the sheath, while detached from the glans clitoris, as in handling, was more voluminous and more hypertrophied than the rest of the organ. The glans clitoris may be also more developed, more prominent; its club-shape, as in the second mode of masturbation, would be in accord with the act of saphism, in which there is not only friction, but also suction of the extremity of the clitoris.

The sad effects of these practices should be combatted with all the resources of therapeutics. I do not believe it necessary to practice cauterization or ablation of the clitoris. We should endeavor to remove the cause which induces patients sometimes to masturbate. Thus, in little girls, the vulvar inflammation should be treated ; in young girls, the oxyuris vermicularis ; in married women, metritis and pruritus vulvæ must be treated, at the same time that the nervous system is acted upon by giving bromide of potassium.

Does prostitution give rise to deformations of the external genital organs ? Authors have different opinions in regard to this. I cannot accept the opinion of M. Charpy, who thinks that the prostitute undergoes in her genital organs a series ef deformations which depend upon the abuse which atrophies and the irritation which hypertrophies. All I can say is, that it appears to me that there exists a peculiar aspect of the genital organs in women, whether prostitutes or not, who abuse coitus.

Thus, in a young girl aged eighteen, deflorated since six months only, and who had never had an abortion and was not pregnant, I found the labia majora enormous, lax, wrinkled, pendant, bluish ; the labia minora were normal, clitoris not developed, and the vulva open. She had metritis, had not had syphilis, and was not addicted to masturbation or saphism.

Struck with this aspect, which resembled rather the genital organs of a woman of forty who had had children, than those of a girl of eighteen, I interrogated her upon her manner of living, and she admitted that for six months she had not passed a single day without three or four sexual connections. These deformities have, then, a value which I cannot disregard, but they do not characterize prostitution ; they may be lacking in the most active prostitutes; while in a young girl they indicate excessive abuse of coitus.

LOCAL MORBID TEMPERATURES IN AFFECTIONS OF THE ABDOMEN.—(*Bulletin de l'Académie de Médecine*, Dec. 9, 1879. By Monsieur Peter.

M. Peter, who has been studying local morbid temperatures for many years, having published articles on that subject as early as 1864, has lately detailed before the French Academy of Medicine four well marked cases of differential diagnosis between ascites and other affections of the abdomen, the diagnosis having been substantiated by post-mortem examinations. The following is a brief summary of those cases. (We would state that M. Peter uses by preference the ordinary thermometer, although he is acquainted with the various supposed improvements involving great cost.)

CASE I.—*Chronic peritonitis distinguished from ascites by elevation of abdominal temperature.* Patient, male, thirty-five years, entered hospital March 25th, affected with abdominal effusion. Had been tapped a few days previous, and seven liters of clean yellow serum withdrawn. He was thin and extremely feeble; skin, dry and sallow (terreuse). Tumefaction of bowels quite marked, the subcutaneous veins slightly enlarged. The liquid moved about easily and palpation produced pain. The liver appeared small from percussion. The tongue was coated, no appetite, with a decided dislike to meat, never any vomiting, no hematemesis.

The disease was of about eight months duration. In consequence of this comparatively rapid development of digestive difficulty the question arose as to whether it arose from an ascites, from cirrhosis of the liver, chronic peritonitis, from chronic gastritis, or cancer of the stomach.

Temperature, March 29th, in axilla, 36.5° over the right hypochondrial region 36.1° (the normal temperature for this region is 35.5.°) That is, in the axilla there was a dimminution of temperature of 0.5°; whilst in the abdominal region there was an elevation of 0.8° above normal, but when compared with the temperature of the axilla, really an elevation of 0.8° + 0.5° or 1.3°. From this elevation M. Peter diagnosticated the difficulty to be not simple ascites, but chronic peritonitis, possibly carcinomatous.

In the early part of April the patient weakened considerably, ate only a few mouthfuls of beef-tea and milk, and that with nausea; he became exceedingly emaciated and the effusion diminished. Temperature on the 11th of April, axilla 36.6°, right hypochondria 36.7.° Died April 25th.

Autopsy revealed: liver small and hard, but not so small as was expected, weight 1250 grams. The stomach was remarkable in apperance; it was small and abounded in ridges (ridé) and completely hidden under the liver. It was somewhat cylindrical in form, bulging out in the middle, and attached to the lower part was an irregular mass about the size of the thumb, of a violet red color, fibroid in character. This was the great omentum, retracted and binding the lower border of the stomach to the transverse colon; the small omentum bound in a similar way the stomach to the liver, and appeared to be equally thick and hard. The peritoneum of the whole region was also chronically inflamed.

The walls of the stomach were at least four times the normal thickness. An histological examination revealed a decided gastric sclerosis.

The ascites then did not arise from cirrhosis of the liver, but from chronic peritonitis, and the local elevation of temperature arose from the inflammation, and not from the ascites.

CASE II. *Chronic peritonitis of tubercular origin, distinguished from ascites by local temperature.* A woman, 33 years, entered the hospital April 4, 1877, with an extensive peritoneal effusion. The diagnosis accompanying her was, "Ascites from cirrhosis of the liver." M. Peter's diagnosis, however, was chronic peritonitis, probably of tubercular origin, without any affection of the liver. The diagnosis was based on the abdominal pain which was present at the commencement of the difficulty, associated with diarrhœa. The thermometer in the abdominal regions gave

| | | | | |
|---|---|---|---|---|
| April | 8th, 36.7°, | 1.2° | above | normal. |
| " | 9th, 36.8°, | 1.3° | " | " |
| " | 10th, 37.4°, | 1.9° | " | " |
| " | 11th, 36.3°, | 0.8° | " | " |
| " | 12th, 36.5°, | 1.0° | " | " |
| " | 13th, 35.5°, | normal. | | |

The 14th, the patient died. The most remarkable feature about the temperature was that on the 12th the axilla gave only 34.5°, and on the 13th, 34.6°. That is, the temperature in the axilla had declined 2° from normal, whilst the temperature of the affected part was one degree higher than normal.

CASE III. *Cancerous peritonitis distinguished from ascites by local elevation of temperature.* Male patient entered hospital June 17 with an accompanying diagnosis of ascites from cirrhosis of liver. The liver appeared small and the spleen large. Gave a history of having vomited blood a year previous, and of having been treated for a difficulty of the stomach. From the time of entering the hospital up to the 20th of June the temperature remained, about axilla, 37°, normal. The effusion increased to such an extent that tapping became necessary, and with the increased effusion came an elevation of temperature, axilla, 38°; abdominal region, 37.8°, or 2.3° above normal.

| | | | |
|---|---|---|---|
| July 6th, | temperature in | axilla, | 38.3°. |
| " 6th, | " | abdominal region, | 38.2°. |
| " 8th, | " | axilla, | 37.8°. |
| " 8th, | " | abdominal region, | 37.5°. |

Just 2° above the temperature for that region.

The patient died August 5th. Autopsy revealed a stomach greatly distended with a cancerous development at the pyloric extremity. The walls near the pylorus were extremely thick. Numerous other pathological changes were present, but our object is simply to show the relation of the elevation of temperature to the cancerous development, as distinct from ascites.

CASE IV. *Chronic peritonitis distinguished from "nervous tympanitis" by local elevation of temperature.* A man of 48 years was presented to M. Peter by a physician of distinction as being the subject of nervous tympanitis. The abdomen was unusually distended, but presented no signs of fluctuation. M. Peter's diagnosis was chronic tubercular peritonitis. The temperature was as follows, taken at different times:

In axilla, 36.9°, over abdomen, 36.6°.
" 36.7°, " 37.1°.

These cases were taken by M. Peter from among a number which he has collected and intends to publish at large. His conclusions are that the simple ascites *never elevates the temperature* of the region; whilst chronic peritonitis *always elevates the temperature.*

A SIMPLE METHOD OF CÜRE FOR OZENA.—(*Gazetta Medica Italiana*, No. 5, 1880, p. 40.)

Dr. Gottestein considers ozena as a constant symptom of chronic coryza. He has no doubt that in consequence of the destruction of the glands, there is is produced a diminution and an alteration of the nasal secretion, which, drying rapidly, remains adherent to the mucous membrane in the form of crusts and undergoes a decomposition which produces the fetor. A limited atrophy of the mucous membrane is sufficient to give origin to the ozena. Adopting this view of ozena, it will be understood that there can be no question of a radical cure, since we cannot hope to establish the normal secretion in an atrophied membrane. We must then seek for a symptomatic cure which should be most simple and least inconvenient for the sufferer.

The author was led to this plan accidentally, while experimenting to see if ozena might be the result of an abnormal dilatation of the nasal fossæ, caused by atrophy of the inferior septa, causing, in consequence, a slowing of the current of inspired airs which is not sufficiently strong to favor the expulsion of the nasal secretions. The method by which the author has been able to obtain perfect results in the space of three months in fifteen case, of ozena is as follows:

He commences with a nasal douche, which removes the secretions from the nasal fossæ and allows him to recognize the state of the mucous membrane and the extent of its lesions. He then introduces into the diseased cavity, a tampon of cotton three to five centimeters in length, which should remain in place for the space of twenty-four hours.

An hour and a half or two hours after its application, patients notice that the nose becomes a little moist. If the tampon be then withdrawn, the mucous membrane is seen to be moist, without crusts or bad odor. An interval of twenty-four hours may elapse between two applications of the tampon. If both nasal fossæ are diseased, the tampon may be used alternately every twenty-four hours. The tampons probably restrict artificially the cavity, which increases the action of the column of inspired air, thus facilitating the expulsion of the secretions; and absorbing continually the matter excreted, prevent its drying up.

PRECOCITY.—M. G. Delaunay, in an interesting paper read before the Société de Biologie on December 27th, records his opinion that precocity is a sign of biological inferiority. As a proof of this, the author adduced the fact that the lower species developed more rapidly and were at the same time more precocious than those higher in the scale. Man is of all animals the longest in arriving at maturity, for his brain may increase up to fifty years of age. The lower races of men are more precocious than the higher, as is seen in the children of the Esquimoes, Negroes, Cochin Chinese, Japanese, Arabs, etc., who are, up to a certain age, more vigorous and more intellectual than small Europeans. Precociousness becomes less and less in proportion to the advance made by any race in civilization. Thus the French people grow less and less rapidly, and to such an extent is this the case, that the standard for recruits has been twice lowered since the beginning of the century; and the same observation applies with equal force to the Italians. In French society the nobles had, according to Broca, in olden times more capacious heads than the rest of the people ; but M. Le Bon now shows that their skulls are at present inferior in capacity to those of scientific men and merchants. Women are more precocious than men, and in all domestic animals the female is formed sooner than the male. From eight to twelve years of age, a girl gains one pound a year on a boy, and in mixed schools they obtain the first places up to the age of twelve. The inferior tissues and organs develop before the higher ones, thus the brain is the slowest of all organs to develop. The anterior and the left superior portion, of the brain, which are the seat of the higher faculties, develop at a later period than the other parts. M. Delaunay concludes by stating that the precocity of organisms and organs is in inverse ratio to the extent of their evolution.—*Le Progrès Médical*, Jan. 3, 1880.

EXPERIMENTAL STUDY ON THE TREATMENT OF HEPATIC COLIC.—M. Laborde. (*Revue Méd.* Feb. 7, 1880, p. 184.)

The following is the author's *résumé* of the results obtained by his physiological experiments concerning hepatic colic :

(1.) The excretory bile canals are endowed with contractile power, and may consequently enter into a spasmodic state under the influence of direct or indirect stimulation; this contractility is of the nature of that of the smooth, muscular fibers of organic life, and the existence of these fibers in the walls of the canals is demonstrated by histological anatomy, perfectly in accord here with experimental physiology. (2.) The mucous membrane of these canals is exceedingly sensitive, and shown under the action of excitants more or less intense by pain and by reflex phenomena, of which the immediate manifestation is spasm of the canals (3.) These phenomena are particularly determined by the presence and contact of foreign bodies (biliary calculi), whose spontaneous migration is thus rendered very difficult and is only accomplished after a greater or less length of time, with the peculiarity that these bodies are carried towards and into the gall-bladder. (4.) Anæsthetic and anti-spasmodic medicines are the most appropriate in the treatment of this morbid state, of which it is easy to realize experimentally the mechanical conditions. (5.) These medicines, notably morphine, chloroform and hydrate of chloral, act by exercising at once an anæsthetic and a paralyzing influence, from which results the cessation of the spasmodic state, the distension of the canals and the accumulation of bile, which acts upon the foreign body as a *vis a tergo* and pushes it toward the intestine. (6.) The combination of chlorhydrate of morphia with chloroform or with hydrate of chloral, that is, the simultaneous administration of these medicines, is the most powerful means of obtaining the desired results, namely: insensibility of the biliary canals, prevention of pain and the favorable influence upon the migration and rapid extrusion of the foreign bodies.

OBSERVATIONS ON THE DIGESTION OF MILK.—Dr. Brush divides milk into two distinct classes, according as it is the product of ruminant or non-ruminant animals. The milk of ruminants contains a variety of casein which coagulates into a hard mass under the action of the digestive ferment, or during lactic fermentation. From this fact the cause of the difficulty experienced by the human stomach in digesting the milk of ruminating animals is readily accounted for. The other variety of milk, viz., that afforded by the non-ruminant animals, does not, under the action of rennet or acid, coagulate into the hard mass which is found in the cow's milk, but forms small granular or flocculent masses which are easily diffusible. This observation explains simply the advantages of koumyss prepared from cow's milk, over the milk itself, in the artificial feeding of children. In koumyss the casein is coagulated, and afterwards subdivided; it is then incapable of further coagulation under the action of any acid or ferment. It is also found that the amount of casein in milk is always in inverse proportion to the amount of sugar present, and the milk of runimants contains a smaller amount of sugar, but a larger amount of casein than that of the non-ruminants. The less sugar too that a given variety of milk contains the more rapidly does lactic fermentation take place, and consequent putrefaction follow. A milk containing a large amount of sugar, however, will undergo alcoholic fermentation, when placed under those conditions which would be the most favorable for lactic fermentation in a milk containing a small amount of sugar. The bearing of this observation is that putrefaction follows lactic fermentation, whereas alcoholic fermentation precludes to a certain extent any form of putrefaction. Thence the value of koumyss in the artificial feeding of children, for the sugar which it contains is all changed into alcohol and its associates, which at this period of life, when properly adminstered, is a true food.—*New York Medical Journal*, Sept., 1879.

CURE OF NASO-PHARYNGEAL POLYPI BY INTERSTITIAL INJECTIONS OF CHLORIDE OF ZINC.—(*Archivio Clinico Italiano*, No. 1, 1880, p. 8.)

Dr. Rochard reported to the *Soc. de Chirurg.* of Paris for Dr. Barthélemy of Toulon, a case of naso-pharyngeal polypus treated successfully with injections of chloride of zinc. The patient was a boy of fourteen years who entered the hospital at Toulon in a state of considerable anemia from abundant nasal hæmorrhages. The polypus had completely destroyed the sense of smell.

Dr. B. proposed several times a radical operation, but the boy refused. He then thought of injecting into the tumor chloride of zinc. As a preliminary, he perforated the palatine arch and took away the most salient portion of the tumor.

The following day he made the first injection of five drops of liquid chloride of zinc. The injections were renewed eight times in the space of four months; at the end of which time the patient was considerably improved and the tumor was almost entirely destroyed. Nevertheless, after some months, a slight relapse was observed in the superior maxilla.

Theophile Anger has twice employed in such cases, with relative success, injections of perchloride of iron, which he believes preferable to chloride of zinc.

TREATMENT OF URÆMIA BY INJECTIONS OF PILOCARPIN.—M. Leven read before the Biological Society of Paris, at its meeting on the 12th of October, an account of a girl, aged 16, who suffered from albuminuria, and who was suddenly seized with convulsions accompanied by complete anuria. Hydrochlorate of pilocarpin was injected subcutaneously, the third injection alone appearing to have any effect. The patient, who had previously been comatose, was then capable of being roused, and she perspired freely, whilst saliva was profusely excreted. The convulsions entirely ceased after the fourth injection, and the patient was cured. During the whole time of the uræmic symptoms the temperature oscillated between 37° and 38° C., whilst the saliva contained nearly ten per cent. of albumen. (*Le Progrès Mèdical*, 25th October, 1879).—*The Practitioner*, February, 1880.

